# Functional miRNA Transfer in Models of Serous Ovarian Carcinoma

**DOI:** 10.3390/cancers18010166

**Published:** 2026-01-03

**Authors:** Goda G. Muralidhar, Hilal Gurler Main, Jia Xie, Joelle S. Suarez, Maria V. Barbolina

**Affiliations:** Department of Pharmaceutical Sciences, University of Illinois College of Pharmacy, 833 S Wood Str., Chicago, IL 60612, USA

**Keywords:** ovarian cancer, miRNA transfer, extracellular vesicles

## Abstract

Background: Intracellular miRNA transfer is an intriguing and lesser-described mode of intracellular communication. Epithelial ovarian carcinoma, of which the high-grade serous histotype represents the most common and deadliest form, is characterized by a microenvironment consisting of tumor and stromal cells, ascitic fluid, and extracellular matrix, presenting a rich milieu of factors that can affect neighboring cells. Methods: We examined the mode of miR transfer in serous ovarian carcinoma cell lines cultured on different extracellular matrix supports both in two-dimensional and three-dimensional formats coupled with traditional, live-cell time-lapse, multiphoton fluorescence imaging modalities, and fluorescence-activated cell sorting approaches. Results: Our data demonstrate that miR can transfer between cells both in culture and in vivo. Moreover, transferred miRNA results in target-specific gene expression changes in recipient cells. Our data indicate that miR transfer occurs via extracellular vesicles, which shuttle from and within the donor and recipient cells via endocytic pathways recruiting sorting, early, late, and recycling endosomes. Conclusions: Our study highlights the phenomenon of miR transfer as a mode of communication between serous ovarian cancer cells, which can affect both treatment and diagnostics of this disease.

## Simple Summary

Cancer cells are regulated by multiple genetic and environmental factors. Due to its specific biology, cells of epithelial ovarian carcinoma are immersed in a rich microenvironment consisting of malignant ascites, stromal cells, parenchymal cells, and their secreted factors, indicating the presence of multiple signaling networks regulating cancer cell fates. A lesser-described mode of intracellular communication in ovarian carcinoma is through the transfer of miRNA. Here we describe that miRNA can be transferred between serous ovarian cancer cells via vesicles, and, once inside the cell, it can initiate transcription of the downstream target genes. Our study also indicates that miRNA taken up by the recipient cell is more likely to be recycled out via endocytosis (as opposed to becoming degraded inside the cell), where it can be absorbed by another cell, thus expanding the range of cells that could be potentially affected by this miRNA.

## 1. Introduction

The incidence of ovarian cancer has been steadily increasing globally over the past century [1]. Epithelial ovarian carcinoma (EOC) remains the leading cause of death from gynecologic malignancies in the US [2]. Most of the patients are diagnosed at late metastatic stages and their chances of survival are low, largely due to the lack of effective anti-metastatic treatments. While the current standard of care, a combination of surgery and chemotherapy, is efficient as an initial treatment, in most cases EOC recurs after a few years, often becoming resistant to treatments, resulting in patient deaths [2,3]. The underlying causes of failure to prolong patient survival stem in part from an insufficient knowledge of basic biology and mechanisms supporting EOC metastasis and therapy resistance.

MicroRNAs (miRNAs or miRs) are a large class of non-coding RNAs that are about 22 nucleotides in length [4]. They are transcribed by RNA polymerases (II and rarely III) to form primary miRNA transcript which is cleaved into pre-miRNA by the action of Drosha [5]. The pre-miRNA is exported to the cytoplasm where it is cleaved by Dicer, and eventually forms the mature single-stranded miRNA [5]. MiRNAs bind to messenger RNAs as part of the RNA-induced silencing complex (RISC) and serve as post-transcriptional regulators of gene expression [6]. They have been identified as key regulators of metastatic progression, tumor response to treatments, and clinical outcomes in EOC [7,8,9]. The miR-200 family members are versatile and important players in EOC [10]. Analysis of The Cancer Genome Atlas revealed that miR-141 and miR-200a, both members of the miR-200 family, are among eight miRNAs that were predicted to be master regulators of at least 89% targets in a miRNA regulatory network characteristic of the pro-malignant phenotype of serous EOC [8]. 

The tumor microenvironment is complex and comprises cancer cells and stromal cells, the extracellular matrix in which they reside, and various signaling molecules secreted by all neighboring cells. The interactions between these components are mediated by different cell–cell, cell–matrix, and signal transduction communication mechanisms. A lesser-described mechanism of intercellular communication involves the cell–cell transfer of microRNAs [11]. It has been reported that miRNAs can be transferred between multiple cell types, including tumor [12]. These miRNAs can be transferred from cell to cell via secreted vesicles known as microvesicles and exosomes [13]. Other modes of transfer, such as those via HDL particles and gap junctions, have been reported as well [14,15]. Multiple studies indicate that vesicles and miRNA found inside them play a role in supporting cancer progression, metastasis, and drug resistance [16,17,18].

Malignant ascites is a unique feature of the EOC microenvironment [19]. The ascites fluid is a rich source of growth factors, cytokines, and other signaling molecules, and it circulates around the abdomen, allowing delivery of its components to the peritoneal tissues and metastatic lesions that seed the peritoneum. Studies have identified exosomes in ascites of EOC patients [20,21]. Exosomes have been suggested to serve as potential messengers for the delivery of functional molecules to the cells [22]. Exosomes from ovarian cancer patients were reported to contain miRNAs, and their delivery to the abdomen prior to the inoculation of tumor cells resulted in an increased tumor load in animal models [23]. These data suggest that the functional transfer of exosomal components, including miRNA, may take place between cells, resulting in dramatic changes in the functional outcomes in the recipients. Such reprogramming of the cell fates in the recipient cells, both tumor and stromal, could result in the development of treatment resistance and aggressive metastatic phenotype among other potential outcomes. Specifically in ovarian cancer, several studies have focused on different types of vesicles (mainly exosomes) and their payloads [24,25,26]. Given their ability to exert powerful effects on proliferation, migration, invasion, and drug resistance, it is understandable that there has been interest in studying the functional role of vesicular miRNA. It may, however, be crucial to expand beyond the functional implications alone. Every novel functional discovery makes it ever more compelling to study the mechanics of the process itself. Hence, we were interested in the characterization of miRNA transfer as a process of mediating intercellular communication. Given its relevance in the pathology of EOC, we selected miR-200a as the candidate to study functional miRNA transfer in EOC.

Several processes have been implicated for their role in the intercellular transfer of miRNAs in different cell models [27]. These include, but are not limited to, gap junctions, apoptotic bodies, HDLs, and intercellular transfer of vesicles. In order to determine which process was largely contributing to intracellular miRNA transfer in the EOC cell lines, we used live-cell imaging with the dual-fluorophore model. We used a time-lapse live-cell fluorescence microscopy approach for constant monitoring of the cells in their typical cell culture environment to observe the mechanism of miRNA transfer. Further, we determined target gene expression in miRNA recipient cells and examined endocytic recycling pathways involved in the transport of transferred miRNA. Our study examines the mechanisms of functional miRNA transfer between cells, thus presenting a way of intercellular communication in high-grade serous ovarian carcinoma.

## 2. Materials and Methods

***Cell Lines***: Human ovarian carcinoma cell lines of high-grade serous histotype Kuramochi and OVSAHO were obtained from the Japanese Collection of Research Bioresources Cell Bank (Osaka, Japan). Human serous ovarian carcinoma cell lines SKOV-3 and OVCAR-4 were purchased from the National Cancer Institute (NCI) Tumor Cell Repository (Detrick, MD, USA). A human high-grade serous ovarian carcinoma cell line, Caov-3, and human ovarian carcinoma cell line ES2 with features of high-grade serous histotype (TP53 mutation [28]) were obtained from Dr. M. S. Stack (University of Notre Dame, IN, USA). Kuramochi, OVSAHO, SKOV-3, Caov-3, and ES2 were cultured according to the manufacturer’s instructions using minimal essential media (MEM) (Corning; Tewksbury, NY, USA) supplemented with 10% fetal bovine serum (FBS) (SIGMA-ALDRICH; St.Louis, MO, USA), 0.5% penicillin/streptomycin (Corning), 0.4% amphotericin B (Corning), and 0.22% g/mL sodium bicarbonate (Santa Cruz Biotechnology; Dallas, TX, USA) for less than 20 consecutive passages. OVCAR-4 was grown in RPMI (Corning) containing 10% FBS, 0.5% penicillin/streptomycin, 0.4% amphotericin B, and 0.2% g/mL sodium bicarbonate. All cells were kept at 37 °C and 5% CO_2_ in a humidified incubator and were routinely tested for Mycoplasma. Cell line authentication was performed for all the cell lines using the STR analysis.

***Materials***. Mirus Label IT^®^ miRNA Labeling kit and mirVana™ miRNA Mimic, negative control #1 (Cat No 4464058), and collagenase type I were obtained from ThermoFisher Scientific (Waltham, MA, USA). Human collagen type I, rat tail collagen type I, and Matrigel were obtained from Corning. Poly-D-lysine was purchased from MP Biomedicals (Solon, OH, USA). pEGFP-N1 plasmid was obtained from Clontech Laboratories (San Jose, CA, USA). ProlongGold was obtained from Invitrogen (Carlsbad, CA, USA). Trizol was obtained from Life Technologies (Carlsbad, CA, USA). Universal cDNA Synthesis kit and ExiLENT SYBR^®^ Green master mix were purchased from Exiqon (Germantown, MD, USA). hsa-mir-200a miExpress™ Precursor miRNA Expression Clone, (Product ID: HmiR0002, precursor sequence: ccgggccccugugagcaucuuaccggacagugcuggauuucccagcuugacucuaacacugucugguaacgauguucaaaggugacccgc) and EndoFectin™ Max Transfection Reagent were obtained from Genecopoeia (Rockville, MD, USA). SV Total RNA isolation system was obtained from Promega (Madison, WI, USA). High-capacity reverse transcription kit and SYBR green were purchased from Applied Biosystems (Waltham, MA, USA). Anti-human EEA1 antibody was from Life Technologies (Waltham, MA, USA), and anti-human-RAB4, -RAB5, -RAB7, -RAB9, and -RAB11 antibodies were from Cell Signaling Technologies (Danvers, MA, USA). Goat anti-mouse-FITC and goat anti-rabbit -Alexa488 antibodies were purchased from Vector Laboratories (Newark, CA, USA). DharmaFECT was from GE Dharmacon (Marlborough, MA, USA).

***miRNA labeling***. Mirus Label IT^®^ miRNA Labeling kit was used to covalently label miRNA mimics with fluorescent tags (either Cy3, used in the imaging experiments, or Cy5, used in the flow cytometry measurements) using the manufacturer’s recommended protocol.

***Transfection of GFP plasmid with EndoFectin™ Max***: The cell lines were transfected with pEGFP-N1 plasmid using the EndoFectin™ Max Transfection Reagent to generate the recipient GFP-positive cell populations for the dual-fluorophore model. The manufacturer’s recommended transfection protocol was utilized.

***Transfection of labeled miRNAs***: The transfection reagent DharmaFECT was used to transiently transfect the labeled miRNAs into the donor cell populations using the manufacturer’s recommended protocol. 

***In vitro dual-fluorophore model to study miRNA transfer using fluorescence microscopy***: Donor cells transfected with fluorescently (Cy3) labeled miRNA mimics were co-cultured with recipient cells tagged with GFP on glass coverslips at 50% confluency. Following 24 h, the cells were fixed with 4% paraformaldehyde solution in phosphate-buffered saline (PBS) and incubated with 4′,6-Diamidino-2-phenylindole (DAPI) at a concentration of 10 μg/mL for 15 min. Cells were washed, air-dried, and mounted on glass slides using ProlongGold. Fluorescence imaging of mounted cells was performed using a Zeiss AxioObserverD.1 fluorescence microscope (Jena, Germany). 

***Live-cell Imaging microscopy of cells cultured using dual-fluorophore model***: Donor cells transfected with fluorescently (Cy3) labeled miRNA mimics were co-cultured with recipient cells tagged with GFP on poly-D-lysine coated Mattek^TM^ glass 35 mm plates at 50% confluency. The cells were imaged using the Olympus Viva View FL Incubator Microscope (Center Valley, PA, USA). Images were acquired in the green, red, and DIC channels every 10 min for 24 h over multiple z-stacks. The acquired images were collapsed and processed using the Metamorph image analysis software (Molecular Devices; San Jose, CA, USA).

***In vivo model to study miRNA transfer using multiphoton microscopy***: 1.5 × 10^6^ donor cells transfected with fluorescently (Cy3) labelled miRNA mimics were mixed with 1.5 × 10^6^ recipient cells tagged with GFP and injected intraperitoneally into athymic nude mice (*n* = 3). After 48 h, the mice were euthanized. The peritoneal wall was excised, and multiphoton imaging was performed using the Prairie Technologies Ultima In Vivo Multiphoton Microscopy System. The 3D and orthogonal reconstruction were performed using the Imaris viewer (Oxford Instruments; Abingdon, Oxfordshire, UK). 

***Coating of cell culture plates with different modified supports***: Tissue culture-treated plates were pre-coated with 10 μg/mL human collagen type I, Matrigel (1:100 dilution), or 0.5 mg/mL Poly-D-Lysine for 1 h at 37 °C. The plates were rinsed with PBS and air-dried before use. 

***Ovarian cancer spheroid models***: The ovarian cancer spheroids were generated by culturing the cells on plates using an agarose overlay method, as described previously [29,30]. The overlay was prepared by pouring 0.5% agarose on the culture plates and then allowing it to solidify and cool at room temperature for at least 30 min prior to adding a suspension of ovarian cancer cells. 

***Flow cytometric quantification of miRNA transfer frequency***: Donor cells transfected with fluorescently (Cy5) labeled miRNA mimics were co-cultured with recipient cells tagged with GFP. After 24 h, the cells were trypsinized, washed, and resuspended in PBS, and then analyzed by using the BD Acuri C6 flow cytometer (Franklin Lakes, NJ, USA). The number of GFP-positive recipient cells that were also positive for the Cy3 miRNA were measured as a percentage of the total number of GFP-positive recipient cells in order to determine the frequency of the miRNA transfer process.

***Measurement of miR-200a expression levels***: miRNA-200a expression levels were determined by qRT-PCR. The miRNA isolation was performed by lysing 1 × 10^6^ cells in 1 mL of Trizol and precipitating with isopropanol and chloroform. Reverse transcription was performed using Universal cDNA Synthesis kit (Roche; Indianapolis, IN, USA) according to the recommended protocol. ExiLENT SYBR^®^ Green master mix was used to perform qPCR for miRNA 200a (Exiqon LNA primer 204539) normalized to controls. UniSp6 and miR-103 were used as normalization controls. Data were analyzed using the 2^−∆∆Ct^ method and Student’s *t*-test, as described previously [31,32].

***Transfection of miRNA Expression plasmids***: The ES-2 cell line was transfected with hsa-mir-200a miExpress™ Precursor miRNA Expression Clone using the EndoFectin™ Max Transfection Reagent to generate the GFP-positive miRNA-200a over-expressing cells, termed ES2-miR200OE. The manufacturer’s recommended transfection protocol was utilized.

***miRNA-200a transfer experimental setup***: The donor cells with high miR-200a expression were co-cultured with recipient cells that expressed little or no miR-200a for 48 h. The donor and recipient cell populations were distinguished by tagging one of the populations with GFP. Using the GFP label, cells were sorted out using the Moflo cell sorter (UIC Flow Cytometry core facility) and the recipient cells were collected for further analysis by qRT-PCR.

***Measurement of miR-200a target gene expression levels***: Total RNA isolation was performed with the SV Total RNA isolation system using manufacturer’s protocol. Reverse transcription was performed using the high-capacity reverse transcription kit, according to the recommended protocol. Q-PCR was performed using SYBR green for *ZEB1*, *ZEB2*, *CTNNB1*, and *TGFβ2* (all predicted gene targets of miR-200a). The primer sequences are as follows:

*ZEB1*

Forward Primer: TTACACCTTTGCATACAGAACCC 

Reverse Primer: TTTACGATTACACCCAGACTGC

*ZEB2*

Forward Primer: AATGCACAGAGTGTGGCAAGGC

Reverse Primer: CTGCTGATGTGCGAACTGTAGG 

*CTNNB1*

Forward Primer: CATCTACACAGTTTGATGCTGCT 

Reverse Primer: GCAGTTTTGTCAGTTCAGGGA

*TGFβ2*

Forward Primer CCATCCCGCCCACTTTCTAC 

Reverse Primer AGCTCAATCCGTTGTTCAGGC

*RPL19* and *EEF1A1* were used as housekeeping gene controls. Data were analyzed using the 2^−∆∆Ct^ method and Student’s *t*-test, as described previously [31,32].

***Gap junction inhibition and scrape-loading dye transfer assay***: 18 Beta glycyrrhetinic acid (18-BGA), carbeneoxonolone (CBX), and 1-octanol were used as biochemical inhibitors of gap junction activity. The cells were treated with the inhibitors (at 50 μM concentrations) in serum-free conditions. Inhibition of gap junction activity was tested using the scrape-loading dye transfer assay. For this assay, the cells were grown in complete monolayers. Following overnight treatment with the inhibitors, the cells were rinsed trice with PBS, and a 0.5 mg/mL solution of Lucifer Yellow was added to the cells. Scrapes were made in the monolayer using a scalpel blade and the plates were left undisturbed, in the dark for 3 min. Cells are then washed with PBS and fixed with 4% paraformaldehyde. The extent of the permeation of the Lucifer Yellow dye from the scrape line into the adjacent cell monolayer was observed by using the Zeiss AxioObserverD.1 fluorescence microscope. The mean fluorescence intensity was quantified using ImageJ (https://imagej.net/ij/; date of access 12 December 2015). 

***Immunofluorescence microscopy***: Donor cells transfected with fluorescently (Cy3) labeled miRNA mimics were co-cultured with recipient cells tagged with GFP on glass coverslips at 50% confluency. Following 24 h, the cells were fixed with 4% paraformaldehyde solution and permeabilized with 0.1% Triton-X-100. Primary antibodies for EEA1 (Life Technologies), RAB4, RAB5, RAB7, RAB9, and RAB11 (Cell Signaling) were used to immunostain for the early, sorting, and late endosomes at a dilution of 1:500 and incubated overnight at 4 °C. Secondary antibodies goat anti-mouse-FITC (for EEA1) and goat anti-rabbit-Alexa488 (for the Rab family) were incubated with the cells at a dilution of 1:1000 for 1 h at RT. Cells were incubated with 4′,6-Diamidino-2-phenylindole (DAPI) at a concentration of 10 μg/mL for 15 min, washed, air-dried, and mounted on glass slides using Prolong Gold (Invitrogen, Carlsbad, CA, USA). Fluorescence imaging was performed using a Zeiss AxioObserverD.1 fluorescence microscope.

## 3. Results

### 3.1. Visualization of Intercellular miRNA Transfer in Serous EOC Cell Lines

**Figure 1:**
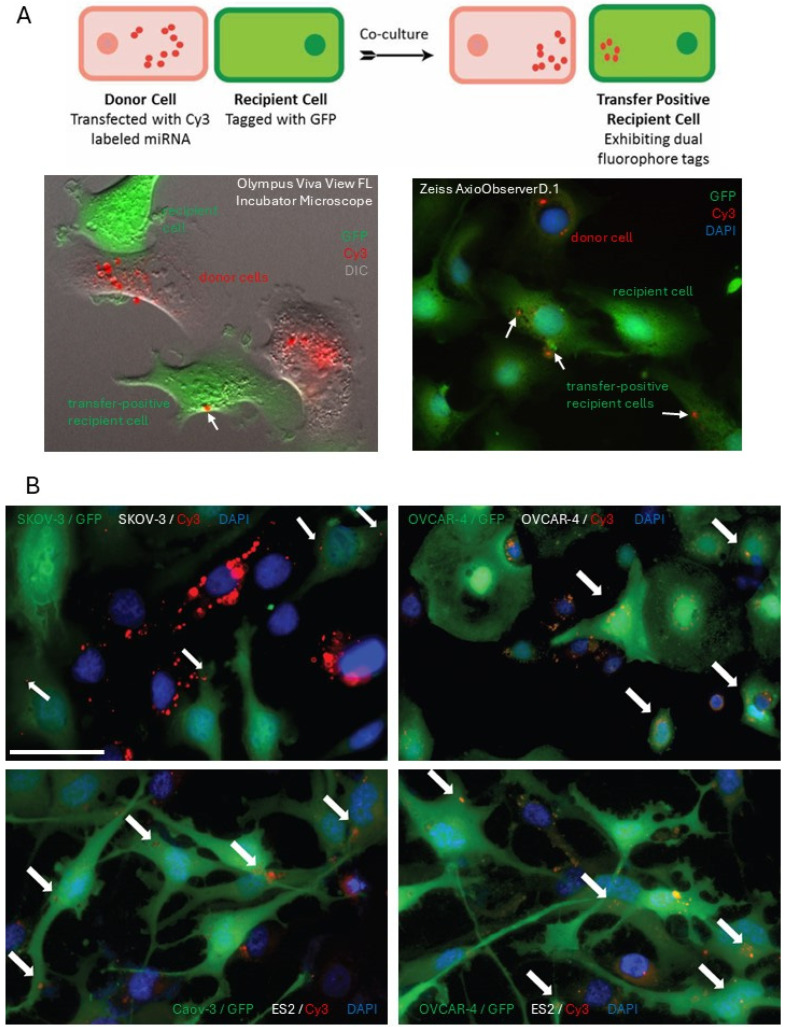
Visualization of intercellular miRNA transfer using fluorescence microscopy. (**A**) A scheme of the dual-fluorophore model used to investigate miRNA transfer. A pink-colored cell represents the donor cell with fluorescently labeled miRNA shown in red puncta. A green-colored cell is the GFP-labeled recipient cell. A green cell with red puncta represents a recipient cell with internalized fluorescently labeled miRNA (termed “transfer-positive recipient cell”). Typical images of the donor, recipient, and transfer-positive recipient cells observed with Olympus Viva View FL fluorescence confocal microscope equipped with a CO_2_ incubator (**left panel**) and Zeiss AxioObserverD.1 fluorescence microscope (**right panel**). GFP-labeled cells were imaged on the green fluorescence channel, Cy3-labeled miRNA were imaged on the red fluorescence channel; blue fluorescence–DAPI. (**B**) Typical images of homotypic and heterotypic transfer of miRNA between serous ovarian carcinoma cells after 48 h co-cultured of donor and recipient cell populations based on at least 5 independent experiments. In the homotypic transfer study, SKOV-3 or OVCAR-4 were GFP-labeled (diffuse green fluorescence; donor cells) and co-cultured with SKOV-3 or OVCAR-4, respectively, with transfected Cy3-labeled miRNA (red puncta; red fluorescence). In the heterotypic transfer study, GFP-labeled Caov-3 or OVCAR-4 (diffuse green fluorescence; donor cells) were co-cultured with ES2 with transfected Cy3-labeled miRNA (red puncta; red fluorescence). White arrows indicate locations of transferred miRNA. Blue fluorescence–DAPI. Bar, 50 μm.

A dual-fluorophore model was established and utilized to observe the transfer of miRNA from one cell to another (Figure 1A). In this model, one cell (termed the “donor” cell) is transfected with red fluorescently labeled miRNA, and another cell (termed the “recipient” cell) is transfected with a GFP-expressing plasmid. Following the co-culture of the two cell types, the transfer of miRNA is visualized using fluorescence microscopy. Two imaging approaches were used: (1) cell were co-cultured for a specified period and then imaged using conventional fluorescence microscopy and Zeiss AxioObserverD.1 fluorescence microscope, and (2) cells were plated together and imaged in 10 min intervals using Olympus Viva View FL incubator confocal fluorescence microscope equipped with a CO_2_ chamber for 24 h (Figure 1A). The transfer of miRNAs was examined between the donor and recipient populations of the same parental cell line (termed “homotypic” transfer), as well as between the donor and recipient populations belonging to different cell lines (termed “heterotypic” transfer). Cy3-labeled control miRNAs were transfected into donor cells and co-cultured with the GFP-tagged recipient cells. To reduce the possibility that the remaining transfection reagent assisted in miR transfer, transfected cells were washed in fresh media trice prior to co-plating with recipient cells. Following 24 h of co-culture, the cells were imaged by fluorescence microscopy. Homotypic transfer of Cy3-miRNA to GFP-tagged recipient cells was observed in SKOV-3 and OVCAR-4 cells. Heterotypic miRNA transfer of miRNA from Caov-3 donor cells to ES2 recipient cells, as well as from OVCAR-4 donor cells to ES2 recipient cells, was observed (Figure 1B). 

**Figure 2:**
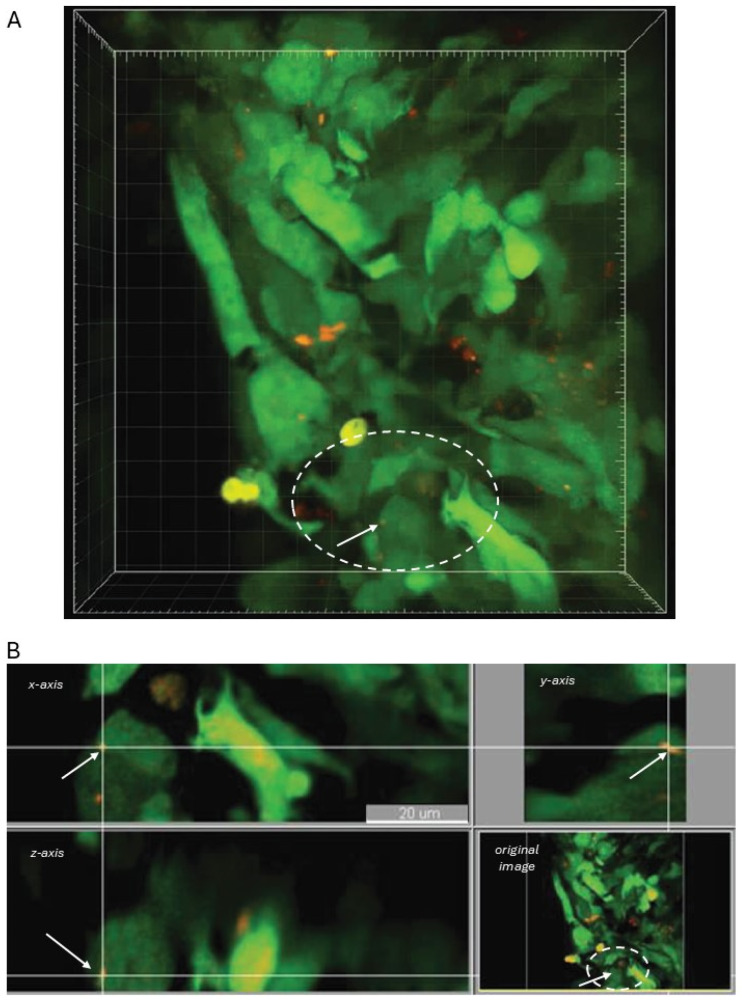
Visualization of transferred miRNA internalized inside the recipient cells using multiphoton fluorescence microscopy. (**A**) Experimental scheme: 1.5 × 10^6^ SKOV-3 donor cells, which are depicted in pink with red fluorescently labeled miRNA were mixed with an equal amount of GFP-labeled SKOV-3 recipient cells and injected intraperitoneally into abdomens of athymic nude mice. (**B**) After 48 h, animals were euthanized, peritoneal walls excised, and imaged using Ultima In Vivo Multiphoton Laser Scanning Microscopy System. A typical image of the three-dimensional reconstruction of a section of the peritoneal wall revealed GFP-tagged recipient cells demonstrating the presence of transferred Cy3-miRNA (red puncta). Bar, 20 μm. (**C**) Orthogonal view of the peritoneal wall section confirmed internalization of the transferred miRNAs in the GFP-tagged recipient cells. The white intersecting axis lines traced over the x, y, and z-axes indicate positioning of the red-labeled miRNA inside the green recipient cell (white arrow) and demonstrate complete internalization of transferred miRNA within the recipient cell. Bar, 20 μm.

These data demonstrated that fluorescently labeled miRNA can transfer from a cell in which it was transfected to another neighboring cell. However, these imaging approaches result in a two-dimensional projection of the cells, thus leaving two important questions unanswered: (1) are miR particles internalized inside the recipient cells or are they adhering to the cell surface and traveling along the surface, and (2) does the miR transfer between cells occur in vivo? To address both questions, we used multi-photon imaging microscopy. A short-term in vivo ovarian cancer cell adhesion was experimentally set up where the donor and recipient cell mixture was injected intraperitoneally (i.p.) into athymic nude mice (Figure 2A). Our previous studies indicated that i.p. injected ovarian cancer cells can adhere to the abdominal wall as early as 4 h post-injection [33]. In this study, we allowed 48 h for cells to adhere to the peritoneal wall and initiate miR transfer. Then peritoneum was excised and imaged using multi-photon microscopy to generate a three-dimensional reconstruction of the tissue (Figure 2B). The reconstruction demonstrated several instances of miRNA transfer like that seen in vitro. The orthogonal view of the imaged section shows the Cy3-miRNA internalized within the GFP-tagged donor cells in the x, y, and z axis (Figure 2C). These results demonstrate that the intercellular transfer of miRNAs between serous EOC cell lines occurs both in vitro and in vivo, and the transferred miRNA is located inside the recipient cells.

### 3.2. Quantification of the Frequency of miRNA Transfer Events

**Figure 3:**
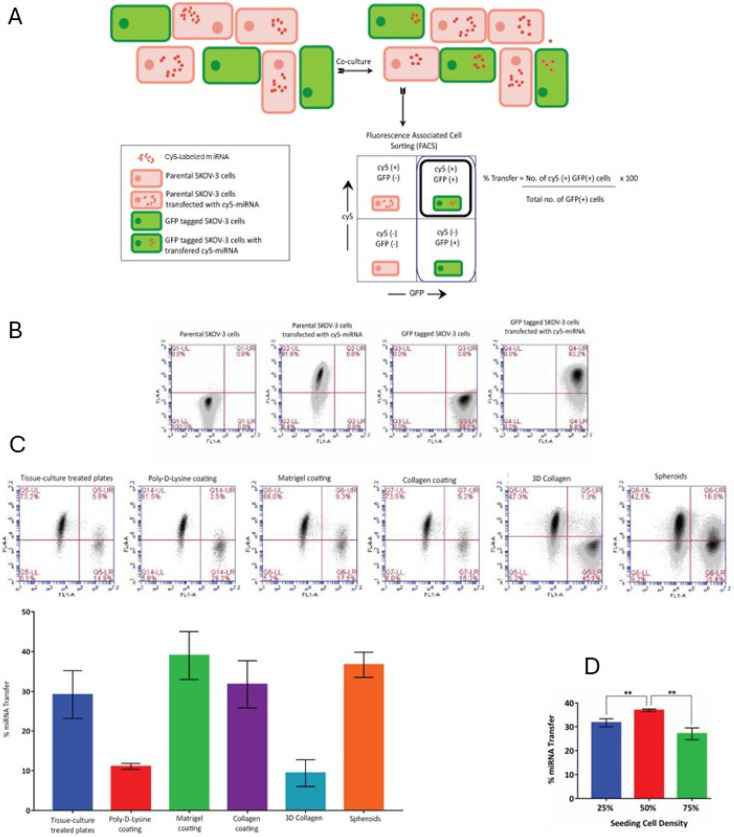
Quantification of the frequency of intercellular miRNA transfer. (**A**) Experimental design to measure the frequency of miRNA transfer by flow cytometry and fluorescence-activated cell sorting (FACS) in conjunction with the dual-fluorophore model described in Figure 1. The donor and recipient cell populations were co-cultured for 24 h. The percentage of transfer was measured as the number of transfer-positive recipient cells (also known as GFP-labeled cells with internalized red fluorescence-labeled miRNA) as a fraction of the total recipient cell population (the number of GFP-labeled cells in the beginning of the experiment) multiplied by 100. (**B**) Representative FACS controls to set the fluorescence thresholds for measurement and analysis for parental unlabeled SKOV-3, unlabeled SKOV-3 with transfected fluorescently labeled miRNA, GFP-labeled SKOV-3, and GFP-labeled SKOV-3 with transfected fluorescently labeled transferred miRNA. (**C**) The percentage of miRNA transfer was investigated using co-culture of Cy5-transfected SKOV-3 and GFP-labeled SKOV-3 in different culture conditions, as indicated, including tissue culture-treated plastic supports, poly-D-lysine coating, Matrigel coating, collagen type I coating, three-dimensional collagen type I gel, and spheroids. Five independent experiments were conducted, and data were averaged and plotted using GraphPad software (Prism7.0). (**D**) The percentage of miRNA transfer was examined in cells cultured on tissue culture-treated supports seeded at 25, 50, and 70% density using co-culture of Cy5-transfected SKOV-3 and GFP-labeled SKOV-3. Three independent experiments were conducted, averaged; data were statistically analyzed using Student’s *t*-test and plotted using GraphPad software; ** *p* < 0.01, Student’s *t*-test.

To quantify the frequency of the miRNA transfer events, we used fluorescence-activated cell sorting (FACS) in conjunction with the dual-fluorophore model (Figure 3A). The miRNA was labeled with Cy5 instead of Cy3. Cells transfected with Cy5-labeled miRNA were termed “donor cell population”, and GFP-labeled cells were termed “recipient population”. Note that potential transfer events between Cy5 miRNA-transfected cells were not accounted for in this model. Cells with transfected Cy5-labeled miRNA were co-cultured with GFP-labeled cells at the final 50% plating density. The percentage of transfer was defined as the number of cells positive for both Cy5 and GFP (also known as the “recipient cells”) divided by the total number of all GFP-positive cells and multiplied by 100. Following 24 h co-culture, the donor and recipient cell populations were sorted out into four sub-populations with distinct fluorescent markers (Figure 3B). Populations of fluorescently labeled and non-labeled cells were used as FACS controls to set the fluorescent thresholds (Figure 3B). 

The process of miR transfer was examined across different cell culture conditions. Cells were cultured on supports typically used for two-dimensional cell culture, such as tissue culture-treated plastic and poly-D-lysine coated plastic, and supports coated with extracellular matrix proteins specific to the EOC microenvironment, including Matrigel and collagen type I. EOC cells are often found in the form of multicellular aggregates or spheroids. When EOC cells adhere to the submesothelial matrix when invading secondary sites, they interact with three-dimensional matrix, predominantly composed of collagens type I and III. Hence, miR transfer between cells in two three-dimensional models were examined as well, including cells cultured as spheroids and atop three-dimensional collagen type I gel (Figure 3C). To examine miR transfer in the context of the three-dimensional collagen culture, cells were seeded atop 0.8% rat tail collagen type I gels. A single cell suspension was prepared by digesting collagen gels with a solution of 5 mg/mL collagenase at 37 °C for 30 min followed by FACS analysis. Cells cultured as spheroids were released into a single cell suspension using 0.25% trypsin/EDTA solution with gentle agitation and analyzed using FACS. We found that miRNA transfer events occurred in all tested experimental culture conditions, both adherent and non-adherent, and were evident in 30–40% of recipient cells in all tested conditions, except for poly-D-lysine-coated supports and 3D collagen type I, where the frequency of miRNA transfer was approximately 10% (Figure 3C). 

We also examined whether the seeding density in the adherent cultures could be one of the factors that determine the efficiency of miRNA transfer. To test the impact of the cell density, the donor and recipient cells were co-cultured at final densities of 25, 50, and 75% on tissue culture-treated supports. The frequency of transfer among these densities was 25–35% and was slightly, but significantly, higher (35%) when cells were cultured at 50% compared to either 25% or 75% plating densities (Figure 3D). Altogether, these results indicate that various culture conditions support transfer of artificially transfected miR in serous ovarian cancer cell lines, although the efficiency of transfer can vary slightly but significantly for cells in different culture conditions.

### 3.3. Functional Transfer of miR-200a in Epithelial Ovarian Cancer Cells

Our data indicated that transfer of an artificially transfected miRNA between cells takes place across different experimental conditions both in vitro and in vivo (Figure 1, Figure 2 and Figure 3). The limitations of the experimental design with an artificially introduced miR created two unanswered questions: (1) is miR transfer a naturally occurring phenomenon or an artifact of experimentally introduced elevated levels of transfected miRNA, and (2) is the transferred miRNA functional, i.e., did it play any role in changing the target gene expression in recipient cells? To address these questions, we constructed experimental conditions in which a gradient of miR-200a expression was created between the donor and the recipient cell populations in both allogenic and syngeneic cell cultures. For experiments with allogenic cell lines, a natural gradient was used where a cell line with little or no basal expression of miR-200a (recipient cells) was co-cultured with a cell line with high basal expression of miR-200a (donor cells). For experiments with syngeneic cell lines, an artificial gradient was created by first over-expressing miR-200a in EOC cells with no basal miR-200a expression (donor cells) and then co-culturing them with parental cell lines (recipient cells). Following co-culture, the recipient cells were collected and analyzed by quantitative RT-PCR (qRT-PCR) to determine whether they contained miR-200a. To determine whether the transferred miRNA was functional in changing the transcription of its target genes in the recipient cells, we determined mRNA levels of selected miR-200a target genes, including *ZEB1*, *ZEB2*, *CTNNB1*, and *TGFB2*. 

**Figure 4:**
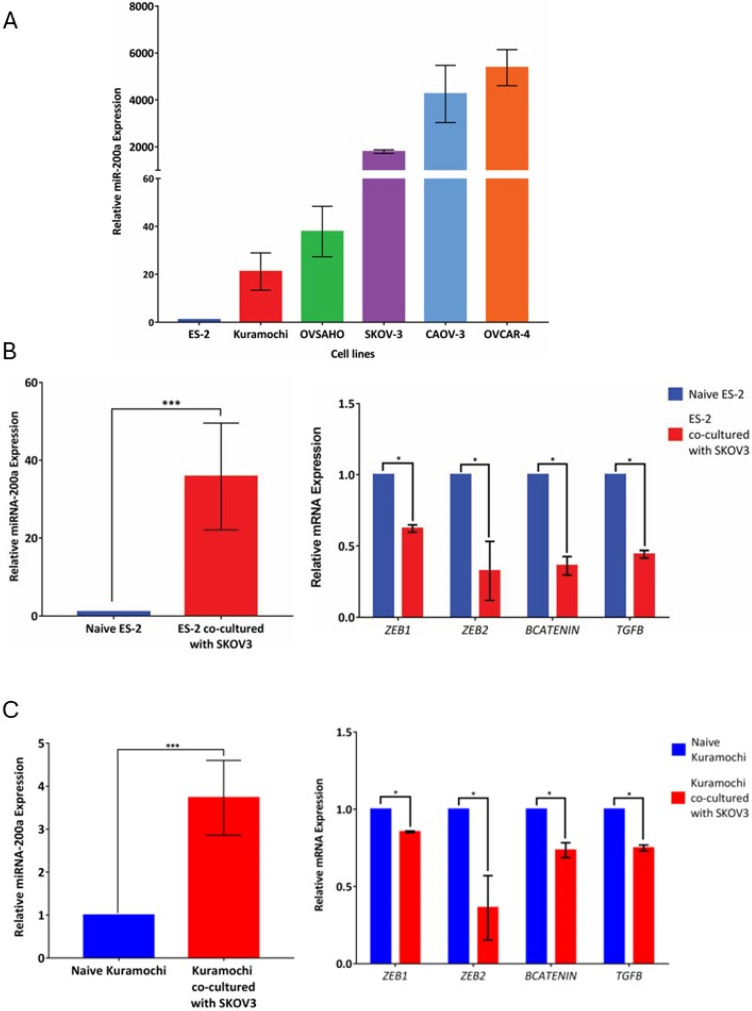
Examination of the functional transfer of miR-200a in serous ovarian cancer cells along the natural miR-200a gradient. (**A**) Basal miR-200a expression in serous ovarian cancer cell lines ES2, Kuramochi, OVSAHO, SKOV-3, Caov-3, and OVCAR-4, as indicated, was measured using qRT-PCR. The data were averaged and plotted using GraphPad software. (**B**) miR-200a transfer across a natural gradient was tested using GFP-tagged SKOV-3 as donor cells and ES-2 as recipient cells. Following 48 h co-culture, ES2 were separated using flow cytometry, and miR-200a expression was measured using qRT-PCR in naïve ES2 and those co-cultured with SKOV-3 cells (**left panel**); *** *p* < 0.001, Student’s *t*-test. Expression of miR-200a target genes, including *ZEB1*, *ZEB2*, *CTNNB1*, and *TGFB2*, was measured using qRT-PCR in naïve ES2 and those co-cultured with SKOV-3 cells (**right panel**); * *p* < 0.05, Student’s *t*-test. Data from three independent experiments were averaged and plotted using GraphPad software. (**C**) miR-200a transfer across a natural gradient was tested using GFP-tagged SKOV-3 as donor cells and Kuramochi as recipient cells. Following 48 h co-culture, Kuramochi were separated using flow cytometry, and miR-200a expression was measured using qRT-PCR in naïve Kuramochi and those co-cultured with SKOV-3 cells (**left panel**); *** *p* < 0.001, Student’s *t*-test. Expression of miR-200a target genes, including *ZEB1*, *ZEB2*, *CTNNB1*, and *TGFB2*, was measured using qRT-PCR in naïve Kuramochi and those co-cultured with SKOV-3 cells (**right panel**); * *p* < 0.05, Student’s *t*-test. Data from three independent experiments were averaged and plotted using GraphPad software.

To test miR transfer across a natural gradient in allogenic cell cultures, the expression of miR-200a was determined in several EOC cell lines by qRT-PCR (Figure 4A). Based on the profiling data, SKOV-3 expressed significant levels of miR-200a, and this cell line was selected as the miR-200a donor. ES2 and Kuramochi had little or no basal expression, and they were selected as miR-200a recipient cells. SKOV-3 (stably tagged with GFP) were co-cultured with ES2 cells for 48 h and sorted using FACS based on the GFP expression. The GFP-negative ES2 cell population was collected and analyzed for miR-200a levels using qRT-PCR. Naïve ES2 cells were used as controls for basal miR-200a expression. We observed that, following co-culture, miR-200a levels in the recipient ES2 cells robustly and significantly increased (Figure 4B). The mRNA expression of miR-200a target genes was significantly downregulated, thereby indicating that the transferred miRNA was functional in changing gene expression in the target cells (Figure 4B). The study was repeated by co-culturing SKOV-3 (stably tagged with GFP) with Kuramochi for 48 h. Similarly, we found that miR-200a levels in Kuramochi after co-culture with SKOV-3 were robust and significantly higher than those in the naïve cells (Figure 4C), and the mRNA levels of miR-200a targets were significantly downregulated as well (Figure 4C). 

**Figure 5:**
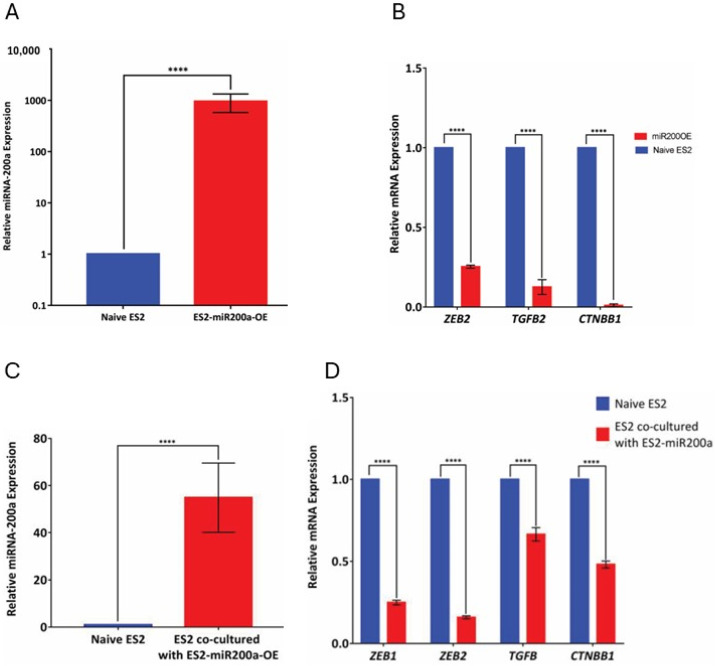
Examination of the functional transfer of miR-200a along an artificial gradient in ES2. (**A**) Basal expression of miR-200a in naïve ES-2 and ES- transfected with miR-200 overexpression plasmid (designated ES2-miR200OE) was measured using qRT-PCR; *p* < 0.0001, Student’s *t*-test. (**B**) Expression of miR-200a target genes, including *ZEB2*, *CTNNB1*, and *TGFB2*, was measured using qRT-PCR in both naïve ES2 and ES2-miR200OE; **** *p* < 0.0001, Student’s *t*-test. (**C**) miR-200a transfer across an artificial gradient was tested using GFP-tagged ES2-miR200OE as donor cells and naïve ES2 as recipient cells. Following 48 h co-culture, ES2 were separated using flow cytometry, and miR-200a expression was measured using qRT-PCR in both naïve ES2 and those co-cultured with ES2-miR200OE; **** *p* < 0.0001, Student’s *t*-test. (**D**) Expression of miR-200a target genes, including *ZEB1*, *ZEB2*, *CTNNB1*, and *TGFB2*, was measured using qRT-PCR in both naïve ES2 and ES2 co-cultured with ES2-miR200OE; **** *p* < 0.0001, Student’s *t*-test. The data from three independent experiments were averaged, analyzed, and plotted using GraphPad software.

For the experiments with syngeneic cell cultures that examined miR-200a transfer across an artificial gradient, we generated ES2 cells that stably overexpressed miR-200 (designated as ES2-miR200OE cells) using a miR-200 overexpression plasmid. Elevated expression of miR-200a in the ES2-miR200OE cells was confirmed using q-RT-PCR with the parental ES-2 cells as controls (Figure 5A). All tested miR-200a target genes were significantly downregulated in the ES2-miR200OE cells compared to the parental ES2 cells (Figure 5B). Then ES2-miR200OE cells were co-cultured with parental ES2 cells for 48 h. ES2-miR200OE cells were tagged with GFP to facilitate sorting of the GFP-negative recipient cells, which were collected and analyzed for miR-200a levels by qRT-PCR. We found that, following co-culture, miR-200a levels in the parental cells increased approximately 50-fold (Figure 5C). Furthermore, mRNA expression of tested miR-200a target genes was significantly downregulated (Figure 5D), indicating that the transferred miRNA was functional in the recipient cells. These data indicate that miR transfer occurs naturally in serous ovarian cancer cell lines, and it plays a role functionally in affecting target gene expression.

### 3.4. Modes of miRNA Transfer in Epithelial Ovarian Cancer Cells

**Figure 6:**
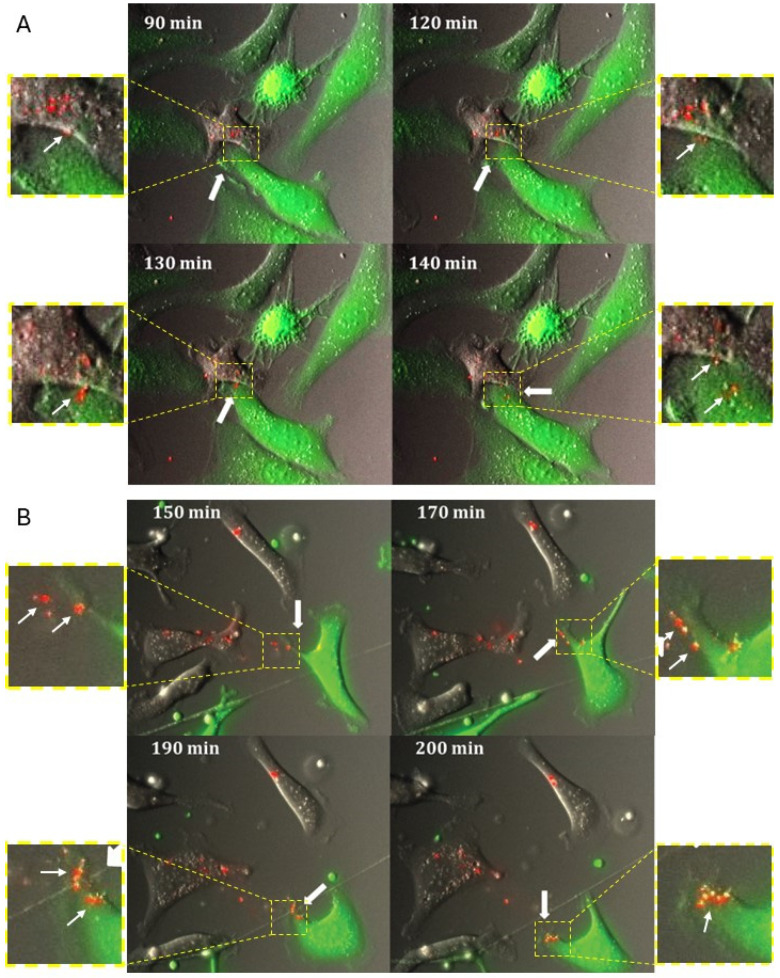
Visualization of mechanisms of intercellular miRNA transfer using time-lapse confocal microscopy. A dual-fluorophore model was used to coculture SKOV-3 with transfected Cy3-labeled miRNA mimic (red puncta) with GFP-tagged SKOV-3. The cells were imaged using the Olympus Viva View FL Incubator microscope equipped with a CO_2_ chamber every 10 min for 24 h on green, red, and DIC channels over multiple z-stacks; the images were collapsed and analyzed using Metamorph software (version 7.8). (**A**) A subset of time-lapse images shows a sequence of events demonstrating passage of a particle-like structure (red puncta) from a donor cell to a recipient cell (diffuse green fluorescence) forming extensive cell–cell contact at the interface of the transfer and maintaining epithelial-like cell morphology. Supplementary Video S1 is an animated version containing this subset of images. (**B**) A subset of time-lapse images shows a sequence of events demonstrating uptake of a particle-like structure (red puncta) from the surface inside the recipient cell (diffuse green fluorescence) maintaining mesenchymal-like cell shape. Supplementary Video S2 is an animated version containing this subset of images. Arrows point at the sites of miRNA transfer or an uptake. Side panels are 3-fold increased images of the areas of interest outlined with yellow dashed lines.

To observe the modes of miRNA transfer in cells cultured in two-dimensional supports, we cultured cells as shown in the dual-fluorophore model (Figure 1) and used time-lapse fluorescence confocal imaging. A time-lapse live-cell microscopy approach allowed for a constant monitoring of the interactions between the cells in their environment. The donor and recipient cell populations were co-cultured on glass bottom plates, coated with either poly-D-lysine or collagen type I, and live-cell imaging performed in 10 min intervals for 24 h. Two major distinct ways of miRNA transfer were observed in our experimental conditions: (1) intercellular miRNA transfer was seen when the cell membranes made close contact with one another, and fluorescently labeled miRNA was seen to shuttle from one cell to another (Figure 6A; Supplementary Video S1), and (2) via an uptake of fluorescent particle-like structures deposited on the supports (Figure 6B; Supplementary Video S2). These observed modes of miRNA transfer from miR-transfected donor cells to GFP-labeled recipient cells have suggested the potential involvement of intracellular and extracellular vesicles and gap junctions as possible mechanisms of transfer.

**Figure 7:**
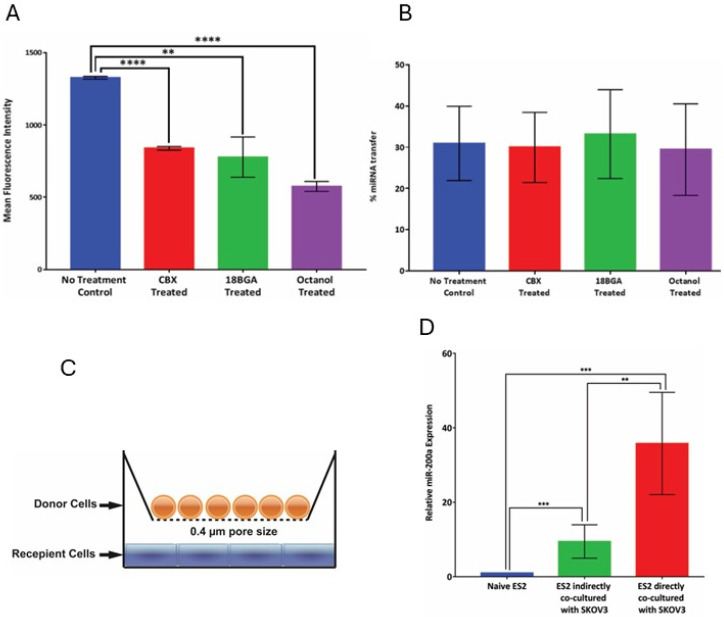
Examination of the requirement for gap junctions for the intercellular miRNA transfer. (**A**) Gap junction activity was examined using pharmacological treatment with 50 µM solutions of gap junction inhibitors carbenoxonolone (CBX), 18-beta-glycyrrhetinic acid (18BGA), and 1-octanol overnight. The gap junction activity was determined by the decrease in the mean fluorescence intensity in treated vs. untreated cell monolayers measured following a scrape-loading dye transfer assay. Data from three independent experiments were averaged, analyzed, and plotted using GraphPad Prism7.0; **** *p* < 0.0001, ** *p* < 0.01, Student’s *t*-test. (**B**) Percentage of miRNA transfer was examined in the presence and absence of gap junction inhibitors. SKOV-3 cell line was cultured as described in the dual-fluorophore model and percentage of transfer was determined using FACS analysis. Data from three independent experiments were averaged, analyzed, and plotted using GraphPad Prism. (**C**) A scheme of the experimental setup to investigate contact-independent miRNA transfer by means of Transwell migration chambers. The donor cells were seeded atop the porous membrane (0.4 μm diameter pore size) filter, and the recipient cells were plated in the bottom of the 24-well tissue culture plate. The filters were placed atop the wells and filled with the conditioned media. (**D**) Comparison of miRNA-200a transfer through contact dependent and contact-independent processes. Mir-200a expression in naïve ES2, ES2 cultured in the bottom of the Transwell chambers, and ES2 co-cultured with GFP-labeled SKOV-3 (as detailed in Figure 4B) was examined using qRT-PCR in three independent experiments, averaged, analyzed, and plotted using GraphPad Prism7.0; *** *p* < 0.001, ** *p* < 0.01, Student’s *t*-test.

We speculated that, if the gap junctions were involved in this process, miRNA could directly shuttle from one cell to the neighboring cell via these channels, which would explain our observations. To test the contribution of gap junctions to the miRNA transfer process, donor cells transfected with Cy3-miRNA were co-cultured with GFP-tagged recipient cells in the presence of gap junction inhibitors; commonly used biochemical gap junction inhibitors 18-beta-glycyrrhetinic acid (18-βGA), 1-octanol and carbenoxolone (CBX) were selected [31,32,33,34]. To verify the inhibition of gap junction activity following treatment with 18BGA, CBX, and octanol, the scrape-loading dye transfer assay was performed on treated cells and untreated controls, and penetration of the fluorescent dye Lucifer Yellow was measured to examine the gap junction channel activity. The assay confirmed that there was a significant reduction (40–50%) in the gap junction activity in the treated cells vs. controls, as evidenced by penetration of the Lucifer Yellow dye into the cells on the edges of the scratch wounds made in the cell monolayers, which was quantified using the mean fluorescence intensity (Figure 7A). Having established the activity of the inhibitors, miRNA transfer was tested in the presence of gap junction inhibition, using the flow cytometry experimental setup previously described in Figure 3A. The efficiency of miRNA transfer was not changed in cells treated with all tested gap junction inhibitors in comparison with the untreated controls, suggesting that gap junction inhibition does not impair the miRNA transfer activity (Figure 7B). However, it remains to be established whether a complete abrogation of gap junction activity is required to rule out this route of miR transfer between cells adopting epithelial morphology.

To determine the contribution of the vesicle-mediated mode of transfer, we set up a contact-independent miRNA transfer experiment to clearly establish a potential role of extracellular vesicles in this process. We constructed an experiment in which the donor and recipient cells were separated from each other but allowed to exchange via common conditioned media. We modified the natural gradient setup to introduce a physical barrier between the donor and recipient cell populations that could selectively allow the passage of vesicles of size < 0.4 μm (Figure 7C). To achieve this, the donor cells with high miR-200a basal expression (SKOV-3) were cultured on Transwell porous membranes (0.4 μm pore size) and the recipient cells with no basal expression of miR-200a (ES-2) were cultured in the lower chamber. In the control experiments, the donor and recipient cells were cultured in the same planar field. Following 48 h of culture, the cells in the lower chamber (experimental conditions) and GFP-negative ES2 (control conditions) were collected and analyzed for miR-200a expression levels. miR-200a was significantly elevated in cells subjected to both the control and experimental conditions, although there was an approximately two-fold higher increase in levels of this miR in cells cultured in the same planar field (Figure 7D). These results suggest that it is likely that most of the transfer events were due to the vesicular uptake from one cell to another. The discrepancies in the observed efficiency of transfer between the Transwell and same planar field experimental setups could be due to multiple factors, including the distance between the donor and recipient cells, dilution, attachment to the porous supports, inhibition of passage of vesicles larger than 400 nanometer, and abrogation of direct cell–cell contact, among others.

### 3.5. Role of Rab GTPases in Transporting Transferred miRNA

**Figure 8:**
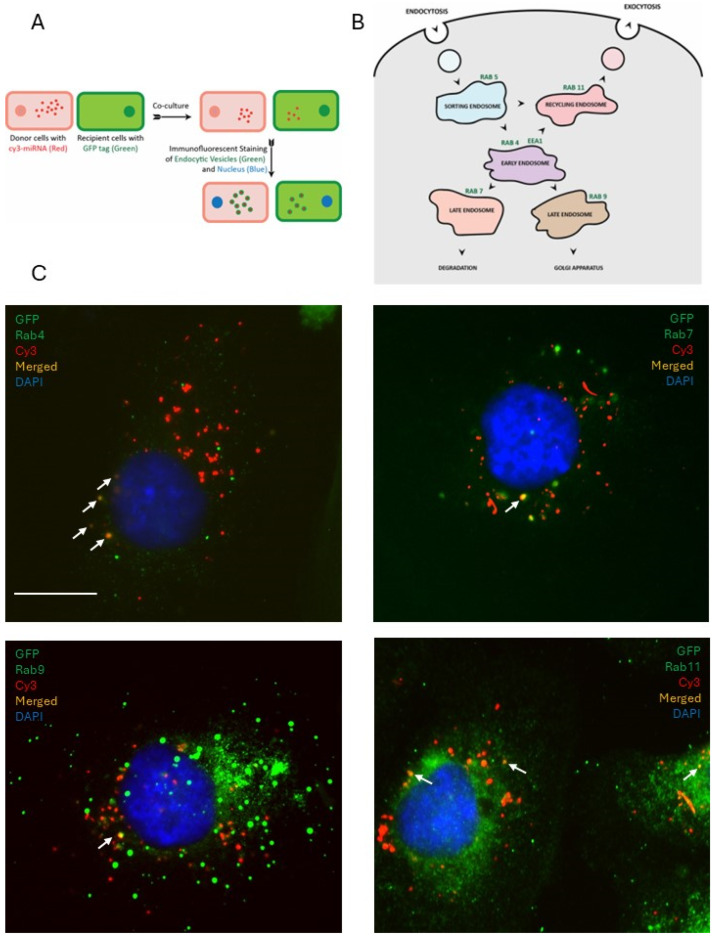
Examination of endocytic packaging of transfected non-functional miRNA mimic in donor cells. (**A**) Experimental scheme for detection of colocalization of transfected miRNA in donor cells and transferred miRNA in recipient cells with endosomal markers. MiRNA is visualized with red fluorescence as red puncta. GFP-tagged recipient cells are visualized by diffuse green fluorescence. Endosomal markers are immunostained and identified with green fluorescence as puncta. (**B**) A scheme of endocytic vesicles and their markers detected in this study. (**C**) Colocalization of transfected miRNA and markers of early (Rab4), late (Rab7, Rab9), and recycling (Rab11) endosomes in donor cell populations was examined using immunofluorescence staining with marker-specific antibodies at 1:500 dilution and FITC-labeled secondary antibodies followed by fluorescence imaging on blue, green, and red channels and superimposition of the images. Arrows indicate endosomes that colocalize with transferred miRNA. Bar, 10 μm.

Having observed the contribution of vesicles to miRNA transfer, we were further interested in characterizing their active transport within the cell along endocytic pathways. We wished to examine the role of Rab GTPases in the transport of vesicles carrying miRNA in the donor and recipient cells. To ascertain whether the miRNAs are processed via endocytic pathways, the following experimental setup was utilized: the donor cells were transfected with Cy3-labeled (red fluorescence) miRNA and recipient cells were tagged with GFP (diffused green fluorescence); endosomal markers were immunostained with specific antibodies and counterstained with FITC- or AlexaFluor488-fluorescently labeled secondary antibodies (visualized as discreet green fluorescent dots) (Figure 8A). Both non-functional miRNA mimic and miR-200a were used in the experiments to enable a comparison between processes regulating their transfer between cells. First, the non-functional miRNA mimic was used in the experiments. Following co-culture for 24 h, markers of early (EEA1, Rab4), sorting (Rab5), late (Rab7, Rab9), and recycling (Rab11) endosomes (Figure 8B) [34,35] were probed using immunostaining and immunofluorescence confocal microscopy in both the donor and recipient cells. 

**Figure 9:**
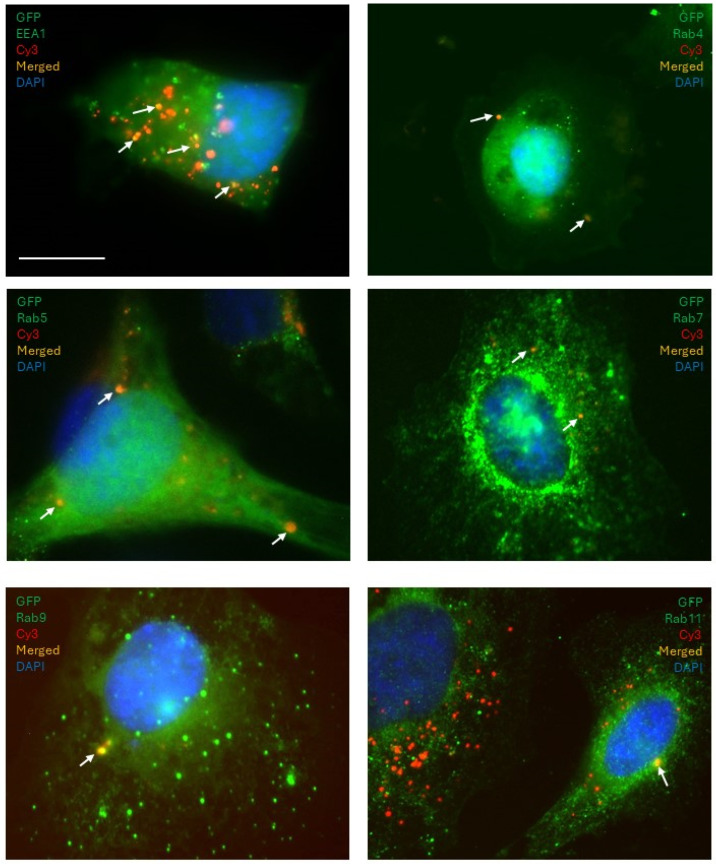
Examination of endocytic packaging of transferred non-functional miRNA mimic in recipient cells. Experimental setup described in Figure 8A was used to conduct the experiments. Colocalization of transferred miRNA and markers of early (Rab4, EEA1), sorting (Rab5), late (Rab7, Rab9), and recycling (Rab11) endosomes in recipient cell populations was examined using immunofluorescence staining with marker- specific antibodies at 1:500 dilution and FITC-labeled secondary antibodies followed by fluorescence imaging on blue, green, and red channels and superimposition of the images. Arrows indicate endosomes that colocalize with transferred miRNA. Bar, 10 μm.

In the donor cells, a fraction of the control miRNA colocalized with markers of early (Rab4), late (Rab7 and Rab9), and recycling endosomes (Rab11) (Figure 8C). In the recipient cells, we observed that a fraction of fluorescently labeled control miRNA colocalized with markers of sorting endosomes (Rab5), early endosomes (EEA1 and Rab4), late endosomes (Rab7 and Rab9), and recycling endosomes (Rab11) (Figure 9). These experiments suggested that the non-functional miRNA mimic, once in the cell, may be processed along different pathways, including becoming recycled back outside the cell, processed in the early endosome and advanced to either Rab7-associated endosome following degradation or to Rab9-associated endosome and transported to the Golgi network for packaging. However, according to our observations, most of miRNA in either the donor or recipient cells was not contained within the Rab-positive vesicles, suggesting that their involvement in the trafficking of the non-functional miR was minor.

**Figure 10:**
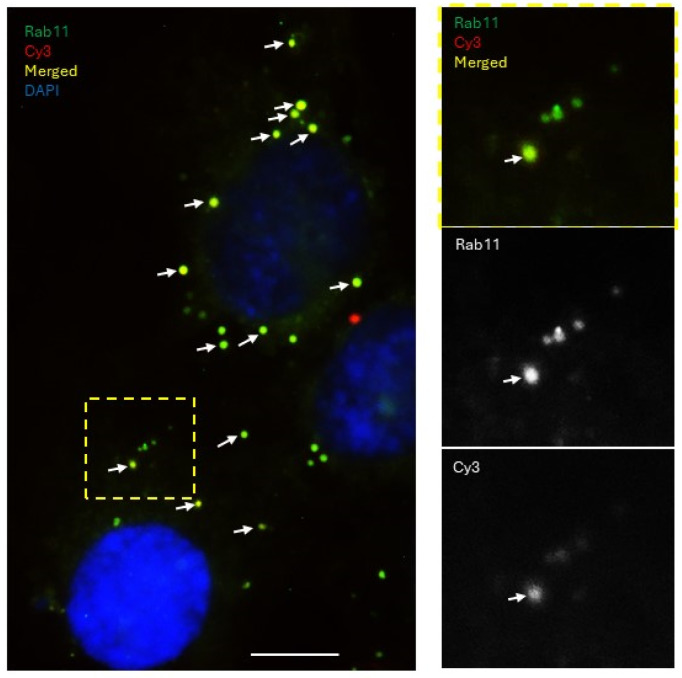
Examination of endocytic packaging of transferred miRNA-200a in recipient cells. Experimental setup described in Figure 8A was used to conduct the experiments. Colocalization of transferred miRNA-200a and a marker of recycling endosomes in recipient cell populations was examined using immunofluorescent staining with Rab11-specific antibodies at 1:500 dilution and FITC-labeled secondary antibodies followed by fluorescence imaging on blue, green, and red channels and superimposition of the images. Arrows indicate endosomes that colocalize with transferred miRNA-200a. Bar, 10 μm. A part of the image outlined with yellow dashed lines was increased twice in size and is shown in the right hand side panels.

Next, to determine whether the circulation of miRNAs through the endocytic pathways depends on its functionality, donor cells were transfected with Cy3 (red fluorescence)-labeled functional miRNA-200a and recipient cells were tagged with GFP (green). Following co-culture for 24 h, the cells were immunofluorescently stained with the recycling endosome marker Rab11 and imaged with fluorescence confocal microscopy (Figure 10). We observed that a predominant fraction of the miRNA-200a was packaged into the Rab11-positive recycling endosomes as compared to its non-functional control counterpart (Figure 9). This may suggest that the cell possesses the ability to distinguish between functional and non-functional sequences and responds to their presence differently using the endocytic pathways. One immediate consequence of shuttling functional miRNA outside the cell may be to share this message with the neighboring cells. More detailed studies of these mechanisms are required to better understand the mechanistic and functional consequences of miR transfer.

## 4. Discussion

The concept of miRNAs acting as mobile signaling molecules is appealing due to their versatility and ability to exert powerful control over the regulation of gene expression. Our findings demonstrate the existence of miRNA transfer between cells in models of serous ovarian carcinoma and indicate the importance of vesicular transport in this process. 

Importantly, our results suggest that the transferred miRNA was functional in affecting the target gene expression in the recipient cells. Epithelial ovarian cancer is known for its heterogeneity in gene expression. In light of our findings, this may indicate that a message in the form of miRNA can easily spread between the neighboring cells in the tumor and its microenvironment and affect target gene expression, thus regulating and contributing to tumor cell heterogeneity. Hence, the relevance of this mode of intracellular communication in this particular malignancy could be very high. Importantly, we observed that miR transfer occurred across different cell culture conditions in which multiple parameters were varied, including plating density, adherent or suspension culture, and materials used as culture supports. This suggested that this mode of intercellular communication could be quite common. Of note, our experimental setup did not include the possibility of quantifying transfer events between the donor cells; in reality, the percentage of miR recipient cells may be higher, if the latter events were also accounted for. 

Our data also indicate that not only the neighboring cells but also those located at a distance from one another can send and receive miRs affecting gene expression in the recipient cell population. It is well established that most ovarian cancer patients develop large volumes of malignant ascites that are also an independent marker of worse outcomes [36,37]. In applying this observation to the situation in vivo, the specifics of the disease pathology suggest that the importance of miR transfer between distant cells in metastatic ovarian cancer can be very high, particularly because the miR-containing vesicles from the donor cells can easily reach many recipient cells by traveling via ascitic fluid that circulates throughout the abdomen. 

Cell lines, primary cultures, multicellular aggregates (for example, mammospheres in breast cancer research and spheroids in ovarian cancer research), tissue explants, and organoids are a mainstay of basic studies of basic biological processes, such as mechanisms of cancer progression and therapy resistance. Our findings may also have direct implications for studies involving cells in culture. Any changes in gene expression due to the up- or down-regulation of expression of a gene of interest, or due to the effects of drugs, may bring about changes in miR gene expression, which may then be transferred to other cells. Even non-proliferating cells may be indirectly affected by drugs through transferred miRs released from cells destroyed by cytotoxic chemotherapy. Depending on the nature of the transferred miR and the target genes it affects, different mechanisms may be up- or down-regulated, resulting in divergent fates in the recipients. Additionally, we found that the efficiency of miR transfer in cells cultured on poly-D-lysine-coated supports and three-dimensional collagen type I gel was lower than those cultured on plastic supports, supports coated with collagen type I or Matrigel, or as three-dimensional spheroids. As poly-D-lysine is often used as a substrate for cell attachment to glass slides for various imaging applications, it is interesting to speculate that the same process that is reliant on miR transfer may be a cause of different experimental results if the gene expression is studied using different cell culture conditions. We have previously described that ovarian cancer cells adopt a pro-metastatic mesenchymal cell morphology when cultured on three-dimensional collagen type I gel [38,39]. This cell type, as opposed to epithelial, does not make extensive cell–cell connections, found to support miR transfer in our experiments. Hence, more research is needed to better understand how changes from epithelial to mesenchymal type may contribute to the cell’s ability to uptake miRNA.

It is interesting to contemplate that the process of miR transfer may be a contributing factor resulting in progression to a more aggressive metastatic phenotype. A recent study found that all miR-200 family miRNAs are overexpressed in the extracellular fraction of the ascitic fluid of HGSOC patients [40]. Thus, miR-200a represented a very attractive candidate for our study, not only due to its consistent involvement in ovarian cancer, but also because of its role in the process of the epithelial-to-mesenchymal transition [10,41,42]. Due to the involvement of the miR-200 family in the EMT transition, the functional transfer of miR-200a could be involved in cell transformation at the secondary tumor sites following metastasis. A double negative feedback loop between miR-200 and the ZEB genes taken into account along with the functional transfer of the microRNA could help us further understand the reversible nature of the EMT process. Intriguingly, attempts to profile miR-200 family expression in EOC [10] produced discordant results, which, to an extent, could be explained by susceptibility to dynamic changes depending on the stage of tumor progression, EMT, localization of interacting proteins to the nucleus or cytoplasm, and cellular ROS content. Functional transfer of miR-200a would add complexity to the mechanisms controlling the fluctuations of its expression and might help in further understanding the variability observed in the profiling studies. Increased levels of miR-200a as well as a decrease in mRNA levels of the target genes provided a phenotypic read-out that coincided with the increased miRNA expression in recipient cells that initially expressed little or no endogenous miR-200a. The conclusion that this increase is due to miRNA transfer is supported by the fact that it was observed consistently over both a natural as well as an artificial gradient in miR-200a expression. The experimental setup with heterotypic cell lines addressed potential cell culture artifacts associated with artificially over-expressing miRNAs to study the process of transfer and demonstrated transfer of miR-200a from SKOV-3 to both ES2 and Kuramochi cell lines. However, more studies are needed to examine whether other miRNAs can also be transferred between cells using the natural gradient, and whether this transfer elicits changes in target gene expression in recipient cells. Importantly, changes in protein levels of the target genes need to be examined along with other functional outcomes, such as the introduction of chemoresistance, changes in cell morphology and EMT, and other processes associated with an increase in disease aggressiveness.

The use of time-lapse fluorescence confocal imaging was very instrumental in visualizing how the transfer of miR can occur between the donor and recipient cells. Intriguingly, two major modes of transfer were observed. In one mode of miR transfer, donor and recipient cells were observed to form cell–cell connections prior to the transfer occurring. It is likely that this mode of transfer may predominate between cells that adopted epithelial morphology. In another mode of miR transfer, dynamically migrating cells on the surface not attached to other cells were observed to pick up and internalize particles containing fluorescently labeled miRs; these particles may have come from multiple routes, such as nontransfected liposomes left over after donor cell transfection, vesicles shed from donor cells, or vesicles and apoptotic bodies left after donor cell death. It is possible that the cells that have undergone mesenchymal transition may favor this mode of miR transfer that is more closely related to endocytic uptake. The first mode of transfer has suggested at least two mechanistic routes of transfer, involving either gap junctions or extracellular vesicles, or both. Using biochemical inhibitors, we found that the contribution of the gap junction channels was insubstantial. However, it is possible that other mechanisms may become activated to compensate for the lack of gap junction activity. The partial restriction of vesicular transport by setting a passage limit to particles under 0.4 μm resulted in a two-fold reduction in the number of miRNA transfer events. However, the transfer still occurred, suggesting that vesicles of multiple sizes both below and above 0.4 μm may be involved in the process. Our study did not focus on detailed description of all possible types of extracellular vesicles that could be involved in this process, and more extensive studies are needed to better describe the types of vesicles that participate in the miR transfer process. It is important to point out that our experimental setup may introduce selection bias, as the liposomal transfection of large amounts of miRNA may preferentially turn on the process of purging the cargo out of the cells, and, in doing so, generate a much larger scale of the transfer. Although this bias was partially addressed with experiments utilizing the natural gradient of miR transfer, more studies of miR transfer involving natural gradients and multiple types of miRs are needed to uncover the scope of the phenomenon. Additionally, yet unknown confounding variables may have their contribution to the outcomes of our study. We attempted to partially eliminate their contribution by using several cell culture models; however, other cancerous and non-cancerous cell models need to be included in the future studies to obtain more generalizable data. 

Imaging experiments to examine the colocalization of the labeled miRNAs with the endosomal markers presented evidence that the miRNAs are, at least partially, shuttled by endocytic trafficking. The sorting and the early endosomes, characterized by the surface presentation of proteins RAB5 (sorting), RAB4, and EEA1 (early), shuttle their contents towards one of three possible fates: (a) degradation in lysosomes through RAB7 late endosomes, (b) to the Golgi apparatus through RAB9 late endosomes, or (c) for extracellular release through RAB11 recycling endosomes. In the donor cells, they were observed packaged into the Rab5-positive sorting endosomes, which then progress into the Rab4/EEA1-positive early endosomes, then maturing into the Rab7/9-positive later endosomes, which were also observed in our experiments. A fraction of the miRNA population was found to colocalize with Rab11-positive vesicles, which can then be released by the cell through exocytosis. These secreted vesicles could then be internalized by the recipient cell and re-enter the endocytic pathway of the recipient cells, thus enabling them to spread the message between cells. Interestingly, we found that the patterns of colocalization of miRNAs with endosomal compartments differed between the non-functional control miRNA and the functional miR-200a mimic. Most of the signal associated with the functional miR-200a mimic colocalized with the Rab11-positive staining, suggesting that miR-200a was predominantly contained within recycling endosomes and was queued up for extracellular release. This presented a scenario in which the distribution pattern through the different endocytic pathways could change depending on whether the miRNA is functional or not. This may suggest that the cells could possess mechanisms that allow them to read and identify a miRNA sequence. This could imply that if the cell receives a message, it is capable of interpreting it and then decide either to act on it (i.e., change the target gene expression), or to ignore it and/or pass it along to another cell. If miRNAs are indeed involved in such a sophisticated system of communication, then the implication would be that the miRNA transfer process is not merely a passive event where the cell discards excess miRNAs into its surroundings where it can be passively absorbed by other cells. It could very well be an active process where a cell intentionally releases certain miRNA-mediated cues which the recipient cells can internalize, process, and decide on how to respond to. This does not exclude the possibility of a “passive” miRNA transfer in addition to or instead of the “active” one. However, if the active transfer does occur, it may indicate the existence of a sophisticated machinery that allows precise communication between an ovarian cancer cell and its microenvironment mediated by miRNAs. Future studies focusing on the examination of how different miRNAs trafficked through the endosomal pathways may help to understand whether such a complicated interaction network exists. Other mechanisms supporting intracellular trafficking, involving exocytosis, endocytosis, and autophagy, need to be examined in the context of miR transfer as well.

There has been considerable interest in using miRNA profiles as clinical biomarkers of various pathologies [43,44,45]. The findings that miRs can transfer between cells may indicate that miR contents in cells could change dynamically based on many factors, which may complicate the use of miRNAs as diagnostic or prognostic markers of the disease progression and therapy response. It is likely that miR contents at any given time in any given cell population reflect just a single pattern in a dynamically changing profile. In future miR profiling studies and gene expression profiling studies, it would be interesting to examine whether miR contents and gene expression change in the same cell population at different time points, as functional transfer of miRNAs could potentially complicate their utility as consistent clinical biomarkers.

## 5. Conclusions

Our study indicates that functional miRNA can be transferred between cells in culture as well as in vivo. This has multiple implications, including better understanding the complexity in the regulation of the gene expression landscape in cancer cells and the potential utility of miR profiling to establish consistent biomarkers of disease or therapy response. Our data indicate the role of extracellular vesicles in the process and suggest that transferred miRNA can affect gene expression in the recipient cells and be trafficked through different endosomal compartments. 

## Data Availability

The original contributions presented in this study are included in the article/Supplementary Material. Further inquiries can be directed to the corresponding author.

## Supplementary Materials

The following supporting information can be downloaded at https://www.mdpi.com/article/10.3390/cancers18010166/s1, Supplementary Video S1: Visualization of mechanisms of intercellular miRNA transfer using time-lapse confocal microscopy in Figure 6A. Supplementary Video S2: Visualization of mechanisms of intercellular miRNA transfer using time-lapse confocal microscopy in Figure 6B.
